# Structural, Compositional, and Plasmonic Characteristics of Ti–Zr Ternary Nitride Thin Films Tuned by the Nitrogen Flow Ratio in Magnetron Sputtering

**DOI:** 10.3390/nano10050829

**Published:** 2020-04-27

**Authors:** Lianlian Chen, Yujing Ran, Zhaotan Jiang, Yinglan Li, Zhi Wang

**Affiliations:** 1 School of Applied Science, Beijing Information Science and Technology University, Beijing 100192, China; chenlianliangf@163.com; 2 School of Physics, Beijing Institute of Technology, Beijing 100081, China; 18810995851@163.com (Y.R.); jiangzhaotan@bit.edu.cn (Z.J.); wlxkwl@bit.edu.cn (Y.L.)

**Keywords:** ternary system, nitride, thin films

## Abstract

Ternary nitride gives high diversity and tunability of the plasmonic materials. In this work, highly crystallized ternary (Ti, Zr)N${}_{x}$ films were prepared by magnetron co-sputtering with different nitrogen gas flow ratio $R_{n}$. The structural and plasmonic properties of the films tuned by $R_{n}$ were investigated. All the films are solid solutions of TiN${}_{x}$ and ZrN${}_{x}$ with a rocksalt structure and (111) preferred orientation. The films are nitrogen-overstoichiometric and the main defects are cation vacancies. Increased $R_{n}$ reduces the zirconium content, and therefore leads to the reduction of lattice constant and enhancement of the crystallinity. As $R_{n}$ increases, the screened plasma frequency decreases for the reduction of free electron density. The maximum of the energy loss spectra of (Ti, Zr)N${}_{x}$ films shifts to long-wavelength with $R_{n}$ increasing. The calculated electronic structure shows that increased nitrogen content enhances the electronic density of states of nitrogen and reduces that of metal, and therefore elevates the energy level at which interband transition is exited. The results show that (Ti, Zr)N${}_{x}$ films give a relatively high plasmonic quality in the visible and near-infrared region, and the film properties can be significantly tuned by the nitrogen content.

## 1. Introduction

For the optoelectronic devices in nanoscale, controlling the nano-dimensional interaction between light and matter has become a major scientific issue and a technological challenge [1,2]. Plasmonics has been regarded as a subject exploiting and controlling the strong interactions between the incident light and plasma oscillations in metallic nanostructures [1,2]. The interaction establishes hybrid modes overcoming the diffraction limit and combines ultrafast and ultrasmall, which is urgent in optical information transmission and processing [1,2,3]. Traditionally, the materials used in plasmonics are noble metals, such as Au and Ag [4,5,6], for their high conductivity in the visible region. However, the large optical losses, chemical and thermal instability, low tunability, CMOS incompatibility, limit their application in plasmonic devices [6]. Requirements for diverse plasmonic materials are now recognized, for a view to integration with semiconductor technology and wide spectral range of plasma resonance.

Among alternative plasmonic materials, transition metal nitride has been proposed as a possible candidate to be used in the visible and near-infrared region [6,7,8,9]. Some metal nitrides have the conductivity comparable to noble metals, and their superior properties of mechanical, thermal and chemical stability bring them potential plasmonic applications in the extreme environment [6]. Furthermore, their wide adjustability in plasmonic characteristics makes them one of the most likely choices for plasmonic application. Titanium nitride and zirconium nitride are the most studied nitrides for its high conductivity, chemical stability and high melting point. The electronic and optical properties of B1-structured nitrides can be tuned by their composition for their nonstoichiometry [10,11,12,13,14]. Recent studies have shown the versatility of nitride plasmonic materials [15]. Many binary [12,16,17] and ternary nitride [18,19,20,21,22] have peculiar plasmonic characteristics, which give a high tunability in plasmon resonance and activation of local plasma behavior. Ternary nitrides were usually obtained by doping TiN${}_{x}$ or ZrN${}_{x}$ with other metal elements. In fact, TiN${}_{x}$ and ZrN${}_{x}$ can be alloyed with each other in any ratio, and the plasmonic properties of their ternary nitride (Ti, Zr)N${}_{x}$ can be modulated by their atomic content in a wide range [20,21,22].

The high tunability of nitrides results from their variable nitrogen and metal content, which is achieved by controlling experimental parameters. For the preparation of nitride films, magnetron sputtering is a usual method [23]. In this work, titanium-zirconium ternary nitride (Ti, Zr)N${}_{x}$ films were fabricated by magnetron sputtering method, and their N content, with a metal content as a consequent, was tailored by the nitrogen flow rate during deposition. The microstructure, chemical state, and dielectric properties of the films were investigated. Different from our previous work [20,21], in which nitride targets were used, in this work metal targets were sputtered, and some experimental results are also different. The electronic band structure of the Ti–Zr ternary nitride was calculated to interpret the results. As expected, the composition, crystal structure and the plasmonic characteristics can be effectively and widely tuned by nitrogen content, but some experimental laws are different from those of the films sputtered from nitride targets. This study demonstrates the potential plasmonic application of this ternary nitride and provides a method for adjusting plasmon performance through a wide range of nitrogen content for the preparation of ternary nitride films by magnetron sputtering.

## 2. Experiments

The (Ti, Zr)N${}_{x}$ films with different compositions were deposited on 10 × 10 × 0.5 mm${}^{3}$ sized JGS1 SiO${}_{2}$ substrates by a magnetron co-sputtering system. All the films were deposited by sputtering a metal Ti (99.9% pure) target and a Zr (99.5% pure) target with two DC power supplies. Both the targets are 3 mm thick and have a three-inch diameter. The sputtered atoms off-normally deposited on the substrate with an incidence angle of about $30^{{}^{\circ}}$. The distance between the substrate and the target is about 130 mm. The background vacuum pressure was 5.0 × 10${}^{-4}$ Pa and the working pressure was kept at 0.8 Pa. Prior to the deposition, both targets were pre-sputtered for 15 min by Ar ions in order to remove the contaminants. Two flows of 99.999% pure Ar and N${}_{2}$ gas were introduced in the vacuum chamber as reactive gases, the substrate rotates 30 revolutions per minute during the deposition to enhance the uniformity of film growth. All the films were deposited at room temperature.

The (Ti, Zr)N${}_{x}$ films were prepared with different nitrogen content, which was tuned by flow rate ratio of $R_{n}$ = N${}_{2}$/(Ar + N${}_{2}$) during deposition. $R_{n}$ was set at 10%, 15%, 20%, 30%, 50%, while the total flow rate of Ar and N${}_{2}$ was kept unchanged at 30 sccm. The sputtering power of both targets was stable at 100 W, and the bias voltage was stable at −50 V. A quartz oscillator was used for real-time monitoring to obtain similar film thickness. The deposition time was about 23–32 min, and the deposition rate was about 6.3–8.7 nm/min. For the films of higher $R_{n}$, the deposition rate is relatively lower and the deposition time is longer.

The crystal structure was characterized by an XRD system (Bruker D8 Advance) with a Cu-Kα1 target (λ = 0.15406 nm). To determine the nitrogen and metal content (N%, Ti% and Zr%), an energy X-ray dispersive spectroscopy (EDS) system (OXFORD, X-act) attached on a scanning electron microscope (Zeiss SUPRA TM 55) was used. In the measurement, we scanned four regions on each film and got the average values as the content. X-ray photoelectron spectroscopy (XPS) analysis was performed on a hemispherical optoelectronic analyzer (Thermo Scientific Escalab 250Xi) with aluminum and magnesium as standard dual anode X-ray sources. The thickness was measured by an XP-1 step profilometer of AMBIOS, and all the films are 200 (±20) nm thick. The study of the plasmonic properties is based on the complex dielectric function characterized by a spectroscopic ellipsometry of Smart SE 850 DUV. The calculation of the electronic structure of the films was independently run through the CASTEP module of the Materials Studio package, and the generalized gradient approximation was performed using the Perdew Burke Ernzerhof function.

## 3. Results and Discussion

### 3.1. Composition and Crystal Structure

The atomic content data of the (Ti, Zr)N${}_{x}$ ternary films are listed in Table 1. Only Ti, Zr, and N are included in the measurement, for SiO${}_{2}$ substrates were used and the measurement of oxygen is difficult. The N content was above 50% for all the (Ti, Zr)N${}_{x}$ films. The nitrogen-overstoichiometry of the films reflects that it is not difficult for ionized nitrogen atoms to combine with metal atoms in a vacuum. It is in the expectation that the N-content of the films increases with $R_{n}$ increasing, which can be explained by the reaction of the nitrogen atom and metal atoms. In the experiments, metal targets were used, and the nitrogen atoms reacting with metal atoms were from the nitrogen flow. Higher $R_{n}$ brought more nitrogen atoms to react with deposited metal atoms and increased the nitrogen content of the films.

Table 1 shows that the Ti and Zr content are also influenced by $R_{n}$. The Ti content is lower than the Zr content in the films deposited with lower (10% and 15%) $R_{n}$s, but it is just on the contrary for the films of higher (20%, 30% and 50%) $R_{n}$s. This result is different from that of the co-sputtered (Ti, Zr)N${}_{x}$ films with nitride targets in our previous work [21], in which Ti atoms prefer lower $R_{n}$s and Zr atoms prefer higher ones. The content of the films is affected mainly by two processes, namely, sputter and absorption. Different $R_{n}$ means a different proportion of nitrogen and argon ions sputtering targets. Argon ions usually sputter metal targets with a higher rate than nitrogen ions, especially for the zirconium target, because zirconium has a higher atomic mass than titanium. So the influence of $R_{n}$ on the sputtering rate of the zirconium target is great than that on titanium target. As $R_{n}$ increasing, the sputtering rate of both the Ti and Zr targets decreases. However, the decrease of the sputtering rate of the Ti target is not as great as that of the Zr target. This is one possible reason why Ti content decrease more slowly than Zr content with $R_{n}$ increasing as shown in Table 1. The absorption and growth process of nitride films in the experiments include the reaction between metal and nitrogen. It is easier for zirconium atoms to react with nitrogen atoms, because their higher electronegativity makes them easier to lose electrons. So the Zr content in the films of lower $R_{n}$ is higher. It is possible that the reaction speed of zirconium with nitrogen tends to saturation when $R_{n}$ is above 15%. The reaction of the titanium gets more benefit from the increase of $R_{n}$, and the reaction speed is accelerated significantly, which also leads to the higher Ti content than Zr content in the films of higher $R_{n}$.

**Figure 1:**
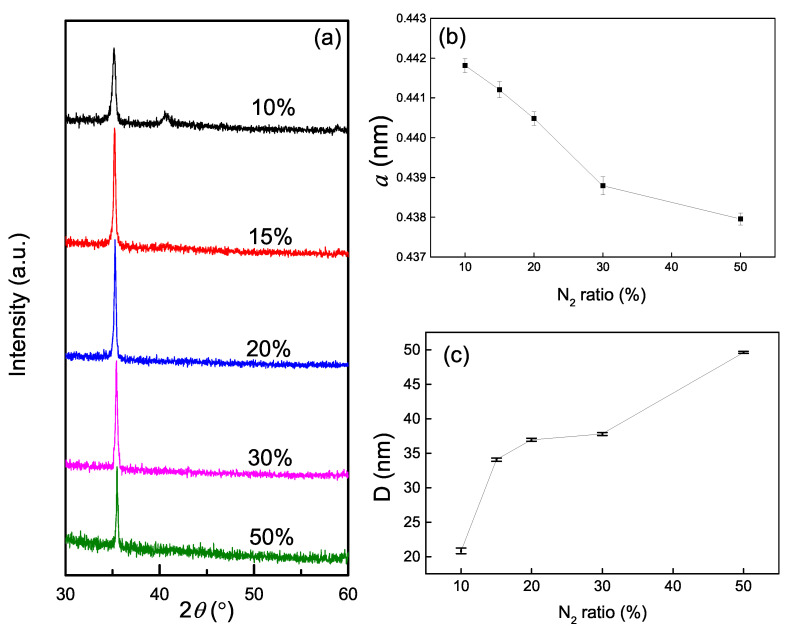
XRD θ–2θ patterns (**a**), lattice constant (**b**), and crystallite size (**c**) of the (Ti, Zr)N${}_{x}$ films deposited with different $R_{n}$.

The variation of the content of the film is also reflected by its crystal structure. Figure 1a shows the evolution of XRD θ–2θ patterns of the films deposited with different $R_{n}$. The analysis of the XRD data was based on JCPDS cards (No. 65-0965 and No. 65-0972). It can be seen for all the films, there is a strong (111) diffraction peak located between the fcc-TiN(111) peak and the fcc-ZrN(111) one. There is also a weak (200) characteristic peak only in the pattern of the films deposited with $R_{n}$ of 5%, which is also between the fcc-TiN(200) peak and the fcc-ZrN(200) one. No other characteristic diffraction peak exists in the patterns. It can be concluded that the films are B1-structured and the preferred orientation is (111), which has growth advantages [10]. The results show that all the (Ti, Zr)N${}_{x}$ films exist as a kind of solid solution, in which the Ti and Zr atoms are distributed in a rocksalt crystal structure with a certain degree of uniformity. Because the Zr and Ti are of the same group and ZrN and TiN are of the same crystal structure, according to the Hume-Rothery rule [24], it is easy to form a solid solution for ZrN and TiN.

As the nitrogen flow ratio $R_{n}$ gradually increases, the (111) diffraction peak moves toward the fcc-TiN (111) peak. The shift of diffraction peak reflects the change of lattice constants. Figure 1b shows the lattice constants *a* of the films calculated by the Bragg equation. It can be easily seen that the lattice constants are in the range of 4.38–4.42 Å and monotonically decrease with $R_{n}$ increasing. Because Ti–N bond is shorter than Zr–N bond, with $R_{n}$ increasing, the increased Ti percent ratio (Ti/Zr), as shown in Table 1, will decrease the lattice constant. Furthermore, the increase in nitrogen content also reduces the lattice constant, though the films are nitrogen-overstoichiometric. As Balasubramanian reported [25], in nitrogen-overstoichiometric B1-structures nitrides, the main defects are cation vacancies, which exhibit negative formation energies, and are thermodynamically favored. While interstitials are difficult to form [25] for their higher positive forming energy. So as $R_{n}$ increases, more and more cation vacancies form, which is another main factor leading to a smaller lattice constant under higher $R_{n}$.

To further investigate the effects of $R_{n}$ on the microstructure of the film, we studied the crystallite size *D* calculated by Scherrer equation,

(1) $$\begin{array}{c} D=0.9\lambda /\beta \cos\theta , \end{array}$$

where β is the full width at half maximum (FWHM) of the (111) peaks value, and θ is the diffraction peak position. The data of the crystallite size are shown in Figure 1c, in which crystallite size increases with $R_{n}$ increasing. This result is different from that of the ternary nitride films co-sputtered with nitride targets [21], in which crystallite size decreases with nitrogen content increasing. For the growth process of films sputtered from metal targets, the chemical reaction is necessary to form nitride film, and nitrogen-rich atmosphere is favored by the nucleation and nuclei merging process of nitride, because nitrogen-rich atmosphere provide much more chance for metal atoms to combine with nitrogen atoms. So higher $R_{n}$ leads to larger crystallite size in this work. However, for the growth process from nitride targets, many nitride particles with raw chemical bonds deposited on substrates, and too much nitrogen possibly leads to more defects in the films. Therefore higher $R_{n}$ leads to small crystallite size in our previous work, in which nitride targets were sputtered.

**Figure 2:**
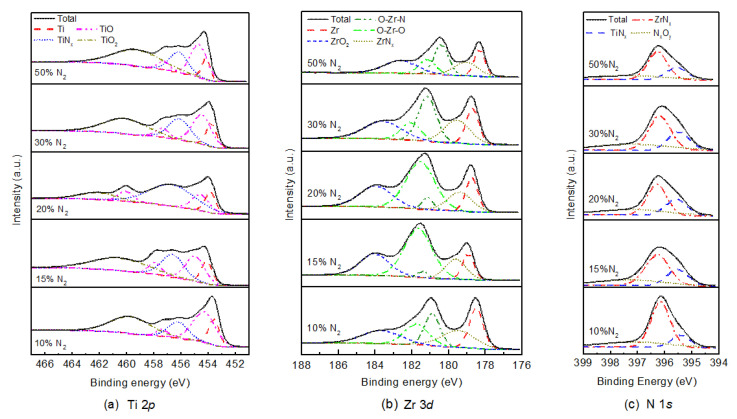
Ti 2*p* (**a**), Zr 3*d* (**b**) and N 1*s* (**c**) XPS spectra of (Ti, Zr)N${}_{x}$ films with different nitrogen content. All samples were surface-etched before testing.

The chemical states of the (Ti, Zr)N${}_{x}$ films were obtained using X-ray photoelectron spectroscopy. All samples were surface-cleaned with argon ion etching of 2000 eV for 56 s before testing. Figure 2a–c shows the XPS spectra of the Ti 2*p*, Zr 3*d* and N 1*s* core energy levels of (Ti, Zr)N${}_{x}$ films, respectively, with the measurement uncertainty below 0.1 eV. The dashed lines are multimodal spectra fitted with Gaussian multimodality, which are used to distinguish different contributions of different bonds. As shown by the dotted line in Figure 2a,b, the characteristic Ti 2$p_{3/2}$ and Zr 3*d* peaks result mainly from the interaction of three kinds of bonds. Besides the expected signals of metal (Ti and Zr) and nitride (TiN${}_{x}$ and ZrN${}_{x}$), there are characteristic peaks of metal oxides. The appearance of the Ti–O, Zr–O, O–Zr–N bonds indicates that oxygen atoms enter some vacancies in the B1-structured nitride lattice, for only the diffraction peaks of rocksalt nitride exist in the XRD results. We think these bonds are related to the residual oxygen or H${}_{2}$O in the vacuum chamber [26]. The peaks near the binding energy of 461 eV in Figure 2a and 183 eV in Figure 2b are almost all contributed by dioxide TiO${}_{2}$ and ZrO${}_{2}$, respectively. It is possible that the titanium dioxide bonds results mainly from the post-deposition oxidization of the films, for there is no diffraction peaks of titanium dioxide in the XRD results after all. Although the plasma etching was performed before the test, it was difficult to eliminate the intended effect of oxygen.

The solid line in Figure 2c shows the N 1s core energy level spectra. Besides a small contribution of the N${}_{x}$O${}_{y}$, the peaks are mainly composed the peaks of TiN${}_{x}$ and ZrN${}_{x}$. Quantitative analysis of integral proportion of the peaks indicates that the integral proportion TiN${}_{x}$ peak gets to its maximum at $R_{n}$ of 20%, while that of ZrN${}_{x}$ peak gets to its minimum at the same $R_{n}$. The variation of the integral proportion reflects the nitrification extend of the metal atoms, which influences the electronic and optical properties of the nitride films.

### 3.2. Dielectric Function

Dielectric properties are fundamental for the study and applications of plasmonic materials. The dielectric functions of the films in this work were measured by incident elliptically polarized light at an angle of 70${}^{{}^{\circ}}$ to an ellipsometry spectrometer. The complex dielectric function ε = $\varepsilon^{\prime }$ + $i\varepsilon^{\prime \prime }$ is determined by the elliptic angles Ψ and Δ, and then fitted to the Drude–Lorentz dispersion model with two Lorentz oscillators. Due to the presence of both intraband and interband transitions in the film, the electronic response in the nitride is fitted using the following equation [27]:

(2) $$\begin{array}{c} \varepsilon =\varepsilon^{\prime }+i\varepsilon^{\prime \prime }=\varepsilon_{\infty }-\frac{\omega_{pu}^{2}}{\omega^{2}-i\gamma_{D}\omega }+\frac{f_{1}\omega_{01}^{2}}{\omega_{01}^{2}-\omega^{2}+i\gamma_{1}\omega }+\frac{f_{2}\omega_{02}^{2}}{\omega_{02}^{2}-\omega^{2}+i\gamma_{2}\omega } \end{array}$$

where $\varepsilon_{\infty }$, $\omega_{pu}$, and $\gamma_{D}$ represent the background dielectric function, the plasma frequency, and the Drude collision frequency (relaxation rate). $\omega_{01}$, $f_{1}$, $\gamma_{1}$ and $\omega_{02}$, $f_{2}$, $\gamma_{2}$ represent the energy position, intensity and broadening parameters of the first and second Lorentz oscillators, respectively. Series of fitting parameters of the film are shown in Table 2.

**Figure 3:**
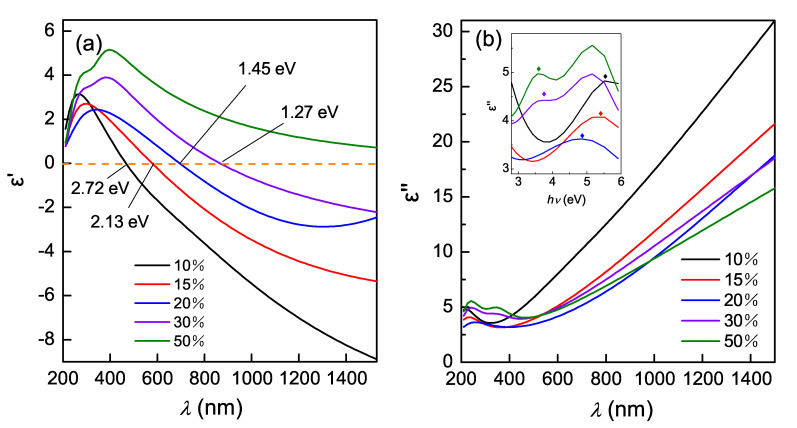
Real part $\varepsilon^{\prime }$ (**a**) and imaginary part $\varepsilon^{\prime \prime }$ (**b**) of complex permittivities of the (Ti, Zr)N${}_{x}$ films deposited with different nitrogen flow ratio. The inset shows the $\varepsilon^{\prime \prime }$ in the near-ultraviolet region, with local peaks marked with diamonds.

Figure 3a,b depict the real part $\varepsilon^{\prime }$ and imaginary part $\varepsilon^{\prime \prime }$ of the permittivities of the (Ti, Zr)N${}_{x}$ films, respectively. As shown in Figure 3a, as $R_{n}$ increases, the $\varepsilon^{\prime }$ curve redshifts. For all the films, as the incidence wavelength increases, $\varepsilon^{\prime }$ decreases from positive to negative values. The positive and negative values of $\varepsilon^{\prime }$ are usually associated with interband and intraband transition respectively. A major practical importance is the crossover frequency $\omega_{c}$, which is defined as the frequency at which $\varepsilon^{\prime }$ = 0. The screened plasma frequency $\omega_{c}$ is associated with the plasmonic resonances frequency $\omega_{pu}$, which is proportional to the square root of the carrier concentration [11]. So the variation of $\omega_{c}$ directly reflects the behavior of carrier concentration. The free electron density *n* can be obtain by Equation (3)

(3) $$\begin{array}{c} n=\frac{\varepsilon_{0}m^{*}\omega_{pu}^{2}}{e^{2}}. \end{array}$$

For the metallic nitride, the effective mass $m^{*}$ of electrons can be approximatively replaced by the mass *m*. The calculated free electron density values were also listed in Table 2.

As shown in Table 2, both the plasma frequency $\omega_{pu}$ and the electron density *n* of the (Ti, Zr)N${}_{x}$ films decreases with $R_{n}$ increasing. The screened plasma frequency $\omega_{c}$, indicated in Figure 3a, is sharply reduced from 2.72 eV to 1.27 eV when $R_{n}$ increases from 10% to 30%. For the films of $R_{n}$ = 50%, $\varepsilon^{\prime }$ is positive in the whole range of the measurement. For ternary (Ti, Zr)N${}_{x}$ film, many factors, such as the inhomogeneity of atomic distribution, point defects of alloying, grain boundaries, possibly trap electrons and reduces the plasma resonance frequency. In this work, it is mainly the increase of N content and the consequent reduction of metal content, as shown in Table 1, that reduce free electron concentration, because the free electrons are gradually absorbed by increasing nitrogen atoms.

The imaginary part $\varepsilon^{\prime \prime }$ of the complex permittivity of the (Ti, Zr)N${}_{x}$ films is given in Figure 3b. In visible range, $\varepsilon^{\prime \prime }$ increases with wavelength increasing. $\varepsilon^{\prime \prime }$ is recognized to be associated with dielectric loss, and the results indicate that in NIR range (Ti, Zr)N${}_{x}$ films of lower $R_{n}$ is more lossy than those of higher $R_{n}$. There are one or two peaks in the $\varepsilon^{\prime \prime }$ spectra in the near-ultraviolet region as indicated in the inset, which is associated with interband transitions [28]. As $R_{n}$ increases, the peak moves towards lower energies. This shift reflects that increased nitrogen content causes the reduction of energy levels at which the interband transition is excited. The other peaks between 5 and 6 eV in the $\varepsilon^{\prime \prime }$ curves of the films deposited with $R_{n}$ of 30% and 50% possibly resulting from the deeper interband transition, which was elevated by increased nitrogen content.

**Figure 4:**
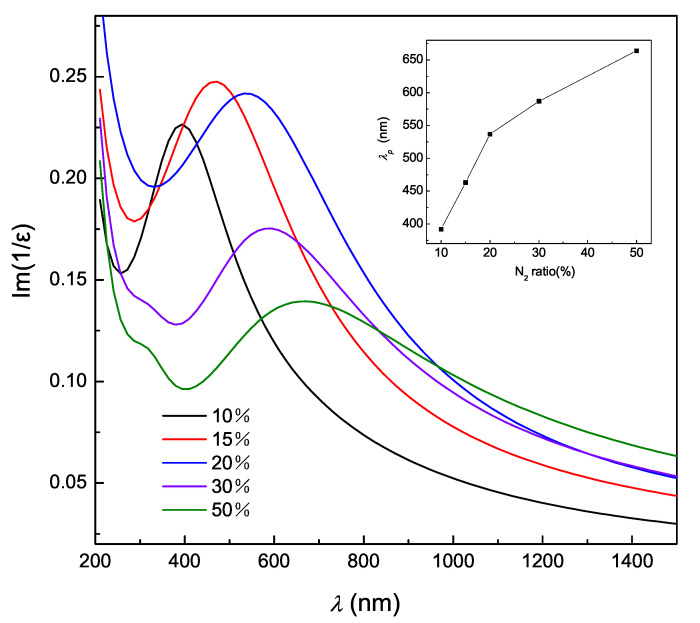
Energy loss spectra of the (Ti, Zr)N${}_{x}$ films deposited with different nitrogen flow ratio. The inset shows the wavelength corresponding to the maximum of the energy loss.

Another parameter for evaluating the plasmonic characteristics of the films is energy loss function, which, are evaluated using equation [29]:

(4) $$\begin{array}{c} -Im\left(\frac{1}{\varepsilon }\right)=\frac{\varepsilon^{\prime \prime }}{{\left(\varepsilon^{\prime }\right)}^{2}+{\left(\varepsilon^{\prime \prime }\right)}^{2}}, \end{array}$$

where $\varepsilon^{\prime }$ and $\varepsilon^{\prime \prime }$ values are the data in Figure 3. Figure 4 exhibits the energy loss function spectrum of (Ti, Zr)N${}_{x}$ films deposited with different $R_{n}$. These spectra indicate the strong plasmon resonance in the films and the effects of $R_{n}$ are significant. With $R_{n}$ increasing, the energy loss peak, shown in the inset, exhibits a red shift. The results also reflect that as $R_{n}$ increases, the metallicity of the film decreases. This experimental law of the energy peak is consistent with that of $\omega_{c}$ in Figure 3a and the peak of $\varepsilon^{\prime \prime }$ in the inset of Figure 3b.

### 3.3. Electronic Structure

The optical and dielectric properties of the films are determined by their electronic structure. For B1-structured metal nitrides, under standard conditions, the difference in the electronegativity between N and metal atoms will cause the metal compound with partial ionicity in the bond. The commingling of metallicity and ionicity is the reason for most of the special characteristics exhibited by metal nitrides. In a close-packed rocksalt structure of nitride, nitrogen atoms occupy octahedral interstitial sites in the (fcc) metal sublattice. In order to obtain the electronic characteristics of Ti–Zr ternary nitride, based on the CASTEP code of the Materials Studio software package, we performed a generalized gradient approximation using the Perdew Burke Ernzerhof function [30,31]. The calculation was based on a primitive cell of B1-structured Ti${}_{0.5}$Zr${}_{0.5}$N, the lattice constant of which is set as 4.40. The cut-off energy of the plane-wave basis set is ultra-fine. The κ-point set was 10 × 10 × 10, and the band energy tolerance and SCF tolerance were 1.0 × 10${}^{-5}$ eV and 5.0 × 10${}^{-7}$ eV/atom, respectively.

**Figure 5:**
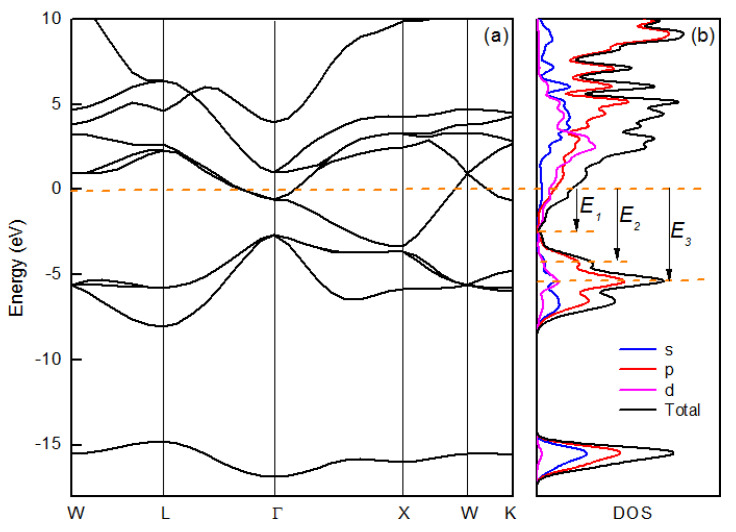
Electronic band structure (**a**) and the density of states (DOS) (**b**) of Ti${}_{0.5}$Zr${}_{0.5}$N.

The calculated electronic band structure and the density of states are shown in Figure 5, in which the Fermi level was set at zero energy. In Figure 5, we identify three separate bundles of energy bands. As indicated in the total and projected density of states (DOS) shown in Figure 5b, each band is composed of a predominant characteristic deriving from a single ionic contribution plus a minor covalent contribution [32]. At higher binding energies around −16 eV, there is a single band with a primary N 2s characteristic. The energy range from −9 to −3 eV is characterized by a bundle of three dispersive bands, which mostly derive from N 2p states partially hybridized with Ti and Zr orbitals. Finally, a bundle of five overlapping bands from −3 to 6 eV around the Fermi level has a main metal characteristic with a weak contribution from N 2p states.

Metallic nitride is not a typical metal, and the presence of interband transition induces small positive $\varepsilon_{1}$ values above the crossover frequency $\omega_{c1}$. The plasmon resonance excitation in the visible range is important for optoelectronic applications, and the origin of this resonance is the interaction between interband and intraband transitions. This can be explained by the electronic structure in Figure 5, in which the band around the Fermi surface is responsible for the metallicity and the negative $\varepsilon^{\prime }$ values in the long-wavelength range. For energies lower than 3.0 eV, the only possible excitations of the valence electrons are due to intraband transitions in Zr 4d or Ti 3d bands crossing the Fermi level [32]. In this range, the partial coupling with N 2p states gives a very low contribution to optical absorption, which leads to small $\varepsilon_{2}$. As the excitation energy is increased, interband transitions from N 2p to Zr 4d and Ti 3d take place. This creates a dielectric screening, which results in zero crossover of $\varepsilon^{\prime }$ from negative to positive at $\omega_{c1}$, at which $\varepsilon^{\prime }$ = 0 and $\varepsilon^{\prime \prime }$ have a minimum. $\omega_{c1}$ is interpreted as a screened plasmon, which involves the collective oscillation of a reduced charge density, because only a fraction of the total valence electrons can be considered as free and the rest are effectively screened [32].

The interband absorption originates from the bands of 3–8 eV below Fermi level. According to the selection rules for photonic excitation, the N 2p electrons in the bands transmit to the Fermi level and exhibit interband transition. As marked in Figure 5, there are 3 characteristic energy levels in the DOS function. $E_{1}$, around 3 eV below $E_{F}$, is the energy level at which Zr 4d or Ti 3d DOS getting to its minimum, and N 2p states begin to dominate the total DOS function. In addition, $E_{1}$ is the cut-off energy level of the N 2p DOS [28], at which the N 2p bands begin to degenerate at Γ point. We found that $E_{1}$ is approximatively equal to the $\hbar \omega_{1}$ value of the films deposited with 10% N${}_{2}$, in Figure 3b. So, $E_{1}$ can be regarded as the threshold of the dielectric contribution to the optical response. The energy level of 4.2 and 5.3 eV below $E_{F}$, marked by $E_{2}$ and $E_{3}$, correspond to a local maximum and the global maximum of the DOS of N 2p bands, respectively. It can also be found that $E_{2}$ and $E_{3}$ are approximatively equal to the positive maximum of $\varepsilon_{1}$ and $\varepsilon_{2}$ in the near-ultraviolet region in Figure 3. The difference between the peculiar energy levels ($E_{1}$, $E_{2}$ and $E_{3}$) in electronic structure and the characteristic frequencies in dielectric function possibly results from the non-stoichiometic defects. Increased nitrogen content enhance the DOS of N 2p band and reduce the DOS of metal, and consequently make the characteristic energy level ($E_{1}$, $E_{2}$ and $E_{3}$ in Figure 5) shift towards fermi level. This shift reduced the frequency of interband transition, at which $\varepsilon_{1}$ crosses zero. The results of the calculation are consistent with the behavior of dielectric function tuned by $R_{n}$ shown in Figure 3.

### 3.4. Plasmonic Quality Factors

The plasmonic performance of a plasmonic materials may be evaluated by factors of merit for localized surface plasmon resonance (LSPR) and for surface plasmon polariton (SPP). The quality factor for LSPR ($Q_{LSPR}$) of nitride particles can be defined as [33,34]:

(5) $$\begin{array}{c} Q_{LSPR}=\frac{\omega \frac{d\varepsilon^{\prime }\left(\omega \right)}{d(\omega )}}{2\varepsilon^{\prime \prime }\left(\omega \right)}, \end{array}$$

where $\varepsilon^{\prime }$ and $\varepsilon^{\prime \prime }$ represent the real and imaginary part of ε in the results above.

**Figure 6:**
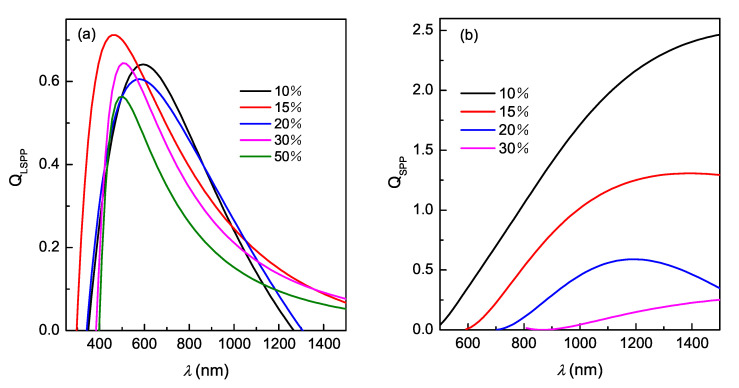
Localized surface plasmon resonance quality factor $Q_{LSPR}$ (**a**), and surface plasmon polariton quality factor $Q_{SPP}$ (**b**) of the (Ti, Zr)N${}_{x}$ films deposited with different nitrogen flow ratio.

The calculated $Q_{LSPR}$ values of all the films are shown in Figure 6a. In the figure, All the films in this study have a relatively high $Q_{LSPR}$, and the strength and location of $Q_{LSPR}$ peaks of the films are different. However, its dependence on $R_{n}$ is not significant, and not monotonous either. This result is different from the $Q_{LSPR}$ behavior of films sputtered with nitride targets, in which increased $R_{n}$ can reduce the value of $Q_{LSPR}$ significantly. The difference possibly results from different portions of the chemical bond in the films. The non-trend resonance peaks in the $Q_{LSPR}$-λ plot indicate the optimal λ region of the corresponding samples for the application in plasmonic devices.

To describe the potential of these nitride films for plasmonic application, we consider the interface of nitride films and air, which sustains SPP formation and propagation. The factor of merit $Q_{SPP}$ of the nitride layers interfaced with air for SPP mode can be given as [33,34]:

(6) $$\begin{array}{c} Q_{SPP}=\frac{1+\varepsilon^{\prime }}{\varepsilon^{\prime }}\frac{{\left(\varepsilon^{\prime }\right)}^{2}}{\varepsilon^{\prime \prime }}. \end{array}$$

The $Q_{SPP}$ spectra of intraband region ($\varepsilon^{\prime }$ < 0) of the films are plotted in Figure 6b. We can see that $Q_{SPP}$ decreases as $R_{n}$ increases for the reduced metallicity of the films. The $Q_{SPP}$ of the films deposited with low $R_{n}$ can be up to 2.5, which leads to a potential application for Ti–Zr ternary nitride as alternative plasmonic materials.

The effects of $R_{n}$ on the plasmonic quality are mainly due to the modification of free carrier concentration by nitrogen content. More and more defects caused by increased $R_{n}$ is another important factor. Nevertheless, the results indicate the feasibility of tuning the plasmonic factors by nitrogen flow ratio.

The plasmonic factors of materials can be affected by many experimental parameters. This work and our previous works [20,21] show that the $Q_{LSPR}$ and $Q_{SPP}$ of the Ti–Zr ternary films are affected by the composition, the substrate bias and temperature during the deposition in magnetron co-sputtering. In these work the peak value of $Q_{LSPR}$ is in the same order of magnitude. In addition, the composition has a greater influence on the shift of the $Q_{LSPR}$ resonance peak. Other parameters have limited influence on the $Q_{LSPR}$ resonance peak position. The peak position of $Q_{SPP}$ is not only related to the composition of the film, but also easily affected by deposition temperature. These works provide a comprehensive reference to select plasmonic materials in specific application scenarios.

## 4. Conslusions

Ternary (Ti, Zr)N${}_{x}$ films were prepared by magnetron co-sputtering with metal targets. The effects of nitrogen gas flow ratio $R_{n}$ on the structural, compositional, plasmonic, and electronic properties of the films were investigated. The results show that B1-structured and (111)-oriented solid solution (Ti, Zr)N${}_{x}$ films were obtained. Not only nitrogen content but also titanium and zirconium content was affected by $R_{n}$, The influence of $R_{n}$ on the zirconium content is greater than that on the Ti content. Increased $R_{n}$ can decrease the lattice constant and enhance the crystallinity for the reduction of the zirconium content and the forming of cation vacancies.

All the films transform from dielectric to metallic with incident wavelength increasing. Increased $R_{n}$ reduces the screened plasma frequency $\omega_{c}$ and the maximum energy in the energy loss spectra of the films. The electronic structure and the density of the state were studied and used to interpret the behavior of the dielectric function. The behavior of the dielectric function is the result of the interplay of interband and intraband transition. The increased nitrogen content causes more cation vacancies, which enhances the DOS of nitrogen and reduced that of metal, therefore elevates the energy level of interband transition, and finally makes the films less metallic and more dielectric.

The results show that high crystallized ternary (Ti, Zr)N${}_{x}$ films with preferred orientation can be obtained with magnetron co-sputtering, and the structural, optical and plasmonic properties of this kind of film can be greatly modulated by the gas flow ratio in a wider range.

## Figures and Tables

**Table 1:** Atomic composition of the (Ti, Zr)N${}_{x}$ films.

N${}_{2}$ Ratio (%)	Ti(%)	Zr(%)	N(%)
10	21.9 (±0.7)	25.9 (±0.7)	52.2 (±1.1)
15	19.8 (±0.7)	22.4 (±0.7)	57.9 (±1.1)
20	19.0 (±0.7)	18.0 (±0.7)	63.0 (±1.1)
30	18.4 (±0.7)	14.8 (±0.7)	66.8 (±1.1)
50	17.0 (±0.7)	13.1 (±0.7)	69.9 (±1.1)

**Table 2:** Results based on Drude–Lorentz fits of the dielectric function nitride films.

N${}_{2}$ Ratio (%)	$\varepsilon_{\infty }$	$\omega_{\mathrm{pu}}$ (eV)	$\gamma_{D}$ (eV)	*n* (10${}^{20}$ cm${}^{-3}$)	$f_{1}$	$\omega_{01}$ (eV)	$\gamma_{1}$ (eV)	$f_{2}$	$\omega_{02}$ (eV)	$\gamma_{2}$ (eV)
10	2.85	6.96	1.51	8.90	0.39	9.65	3.63	0.45	3.15	2.38
15	2.73	5.79	1.85	6.16	1.72	2.73	1.64	0.39	8.95	3.39
20	3.17	5.65	2.17	5.86	0.37	9.01	3.08	1.40	3.02	1.44
30	2.01	5.12	1.27	4.82	0.38	9.53	4.33	0.14	3.60	3.45
50	1.60	4.24	1.81	3.30	0.06	2.74	0.98	0.32	10.26	5.85
